# Lipofibromatous hamartoma of a digital branch of the median nerve: A case report and review of literature

**DOI:** 10.1002/ccr3.4728

**Published:** 2021-08-30

**Authors:** Jorge Barraza‐Silva, Roberto Berebichez‐Fridman, Lilia Edith Corona‐Cobian, Laura Montserrat Bernal‐López, Raúl Álvarez‐San Martín

**Affiliations:** ^1^ Orthopaedic Surgery Department American British Cowdray Medical Center Mexico City Mexico; ^2^ Pathology Department American British Cowdray Medical Center Mexico City Mexico

**Keywords:** lipofibromatous hamartoma, macrodactyly, median nerve, median nerve tumor, nerve compression, peripheral nerve

## Abstract

Lipofibromatous hamartoma (LFH) is a benign tumor that causes nerve enlargement due to fatty adipose tissue infiltration around bundles of peripheral nerves. It most commonly occurs at the median nerve with associated macrodactyly. We present an uncommon case of LFH that affected a digital branch of the median nerve without macrodactyly.

## Graphical abstract

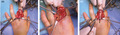

## 1 INTRODUCTION

Lipofibromatous hamartoma (LFH) is an uncommon benign tumor that causes nerve enlargement due to fatty adipose tissue infiltration around bundles of peripheral nerves. Its clinical presentation may vary, but its main symptoms are associated with neural compression, and the most commonly affected site of LFH is the median nerve. Its diagnosis can be confirmed after observing pertinent changes in ultrasonographic and magnetic resonance imaging (MRI), but when in doubt, a biopsy of the lesion is needed.^1^, ^2^ Due to the lack of clinical studies, there is currently no standardized treatment for LFH. Therefore, observation, carpal tunnel release, nerve decompression, or excision of the lesion can be performed.^1^, ^2^ We report a case of an 18‐year‐old female patient with LFH at a digital branch of the median nerve of the middle finger of the left hand and a review of current literature on LFH.

## 2 CASE REPORT

An 18‐year‐old woman without any medical condition or genetic disorder presented after a fall from a horse. She had forced traction of the middle finger of the left hand from the reins of the horse.

The patient presented with insidious burning pain and numbness in the dorsal part of the proximal interphalangeal joint of the middle finger. No fractures were seen on radiographs; therefore, analgesic treatment was provided. After 4 months, pain remained constant and worsened with direct pressure of the proximal interphalangeal joint. She denied having fever or weight loss.

**FIGURE 1:**
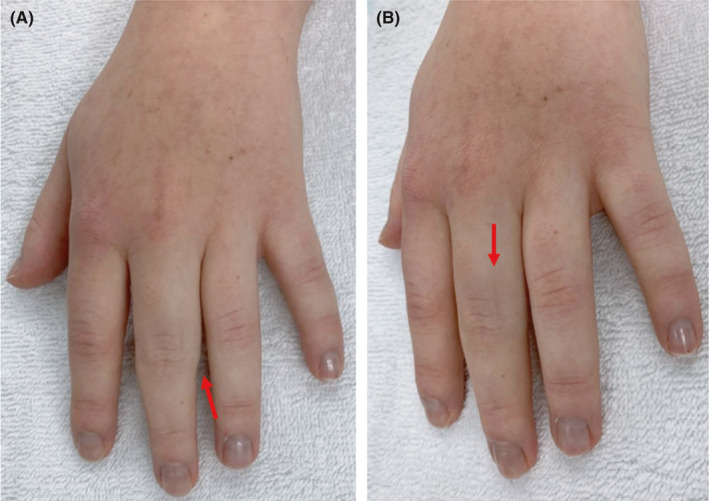
A, Diffuse mass (approximately 1 × 1 cm) on the ulnar and dorsal side of the proximal phalanx of the middle finger of the left hand. B, Purple color of the proximal interphalangeal joint

Physical examination revealed a diffuse mass (approximately 1 × 1 cm) on the ulnar and dorsal side of the proximal phalanx of the middle finger of the left hand. The mass was not adherent to the skin and was mobile (Figure 1). The range of motion of the metacarpophalangeal joint was 90° of flexion and 0° of extension. The range of motion of the proximal interphalangeal joint was 15° of flexion and 0° of extension. The range of motion of the distal interphalangeal joint was 20° of flexion and 0° of extension. She had pain upon palpation and was positive for Tinel's sign on both the ulnar and dorsal sides of the proximal phalanx of the middle finger. Hyperthermia, skin changes, and palpable lymph nodes were not reported. Sensitivity was normal, and there were no paresthesias observed.

**FIGURE 2:**
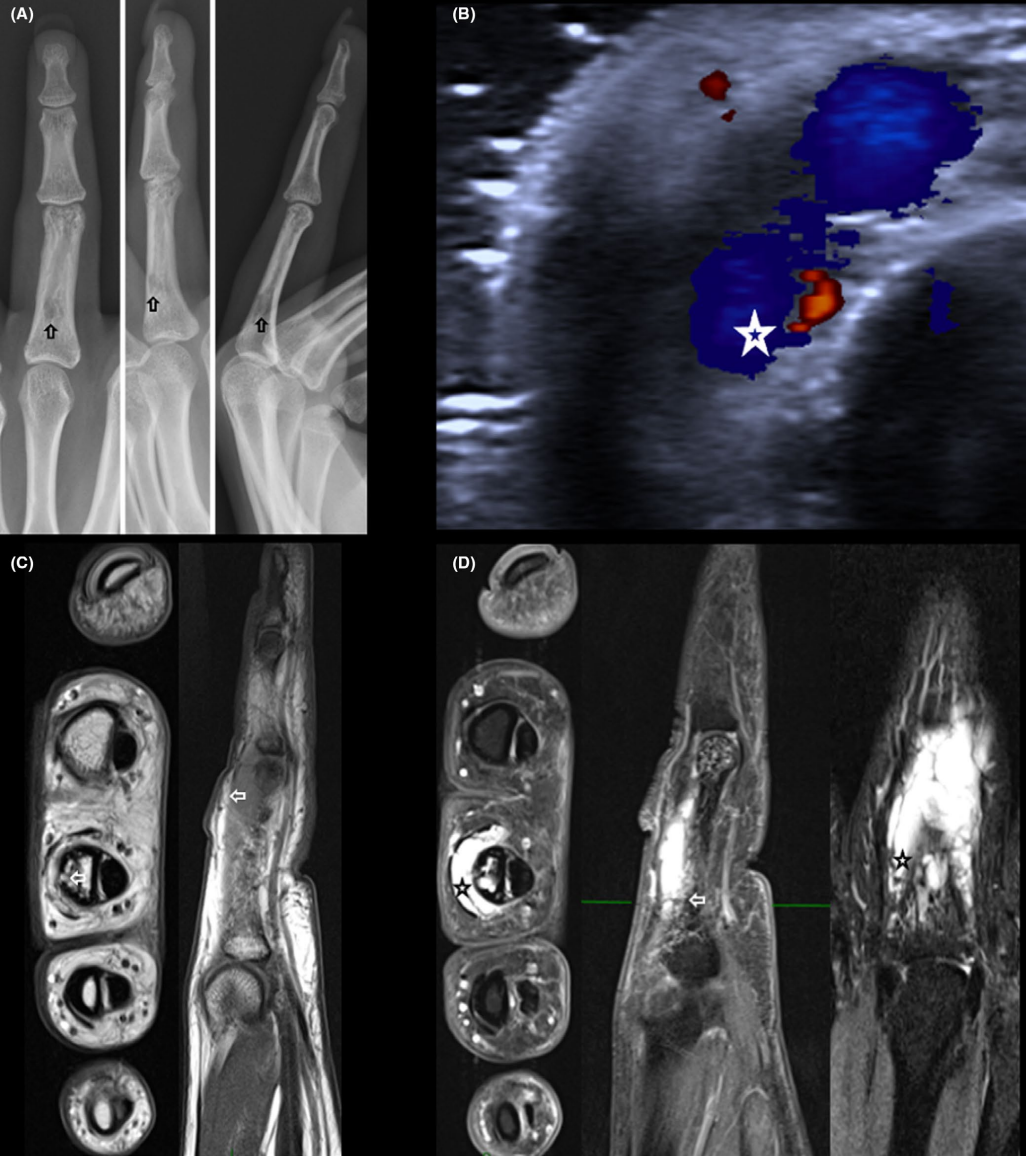
A, Anteroposterior, lateral, and oblique radiographs of the middle finger showing a circular radiolucent image with thinning of the posterior cortical area (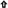). B, Ultrasound of the middle finger. Dorsal side of the middle finger with ectasial venous flux communicating with an arterial branch can be seen (*). C, MRI of the middle finger without gadolinium. A 36 × 5 × 11 mm tumor located on the dorsal aspect of the proximal interphalangeal joint, which displaced the extensor tendons, can be seen (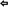). D, MRI of the middle finger after gadolinium administration. The lesion enhanced, and there is a 1‐mm focal discontinuity of the cortical bone seen (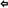) with one ectasical vascular trajectory (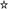).

Radiographs were performed, which showed a circular radiolucent image measuring 6.5 × 5 mm with thinning of the posterior cortical areas of the proximal phalanx of the middle finger (Figure 2A).

Ultrasound of the finger was performed, which showed vascular trajectories with ecstatic flux and venous morphology. Communication with an arterial branch was observed, which condition cortical bone irregularities (Figure 2B).

An MRI was performed, which showed a 36 × 5 × 11 mm lesion located on the dorsal side of the proximal phalanx, displacing the extensor tendons. The lesion was enhanced after gadolinium administration. There was a focal discontinuity of the cortical bone of 1 mm observed with an ectasical vascular trajectory (Figure 2C,D).

An incisional biopsy of the bone and soft tissue using a Jamshidi needle was performed. This illustrated a benign lesion constituted by fibrofatty tissue.

**FIGURE 3:**
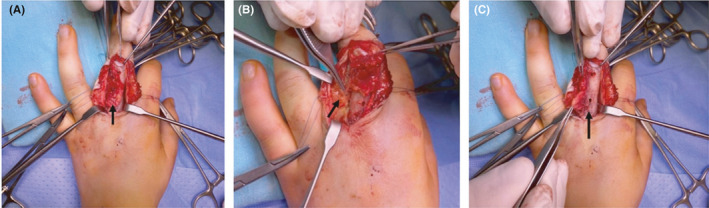
A, A 4.3 × 3 × 0.4 cm irregular and gray tumor located on the ulnar side of the proximal phalanx of the middle finger. B, Tumor within the neurovascular bundle of the ulnar side of the proximal phalanx of the middle finger. C, Gray‐like color on the dorsal part of the proximal phalanx and a 1‐mm focal discontinuity of the cortical bone

Definitive surgery was done 2 weeks later via complete excision of the lesion. We debrided and resected an irregular and gray tumor of 4.3 × 3 × 0.4 cm (Figure 3A) located on the neurovascular bundle of the ulnar side of the proximal phalanx of the middle finger (Figure 3B). The bone had a gray‐like color on the dorsal part of the proximal phalanx and a 1‐mm focal discontinuity of the cortical bone (Figure 3C). A 20 × 10 mm window was created to observe the medullary canal, which had a yellowish and gray‐like tissue that was removed. The medullary canal was obliterated in the proximal region. Finally, the bone window and surgical wound were closed.

**FIGURE 4:**
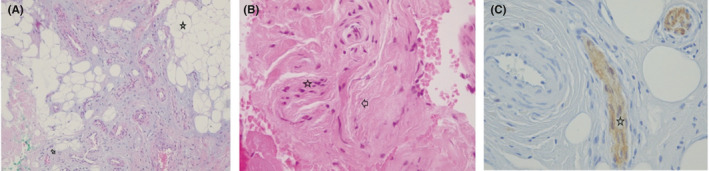
A, HE 4×. Histopathological findings. There is fibrofatty tissue and connective proliferation (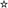) compressing nerves bundles (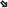). B, HE 40×. Histopathological findings. Nerves fascicules (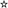) compressed by overgrowth of connective tissue (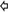). C, S100 immunohistochemistry highlights the Schwann cells in nerve fascicules (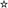)

Histopathological examination showed a benign lesion constituted by fibrofatty tissue that compressed the nerve fascicules with overgrowth of the remaining connective constituents, proliferation of blood vessels, myofibroblastic proliferation, and collagen deposition (Figure 4A,B). Immunohistochemistry was performed with S100, which highlighted nerve fascicles compressed by the tumor. Neither necrosis nor atypical mitosis was observed (Figure 4C).

At the latest follow‐up (7 months), the patient had no pain at the surgical site. The range of motion of the finger was preserved. She had no paresthesias but continued to have numbness on the dorsal part of the proximal interphalangeal joint of the middle finger.

## 3 DISCUSSION

Lipofibromatous hamartoma is an uncommon tumor that affects the peripheral nerves wherein fibroadipose tissue infiltrates the epineurium and perineurium.^1^ Its most commonly presents in the 30s–40s, and the median nerve is affected in 80% of the cases. However, other nerves can be affected, including the brachial plexus, cranial, ulnar, radial, sciatic, peroneal, plantar, and digital nerves.^2^, ^3^ Its signs and symptoms generally depend on the location of the tumor. It can present as a compressive neuropathy, which may have associated sensory and/or motor deficits on nerve distribution. Pain and tenderness may also be associated.^2^ One third of the cases are associated with macrodactylia.^4^

In 1953, Mason^5^ first described LFH. In 1985, Silverman and Ezinger studied 26 cases of LFH in which the main age distribution was between the 3rd and 4th decades of life. There were 25 cases located on the wrist and hand, and only one case was found on the toe.^6^ Although it most commonly affects Caucasian males, LFH with associated macrodactylia is most commonly seen in females.^7^ Generally, macrodactyly is a common finding in 20%–66% of cases.^8^, ^9^

The cause of abnormal growth of the nerve remains unknown.^2^ It is also unknown why the median nerve is most commonly affected.^7^ Some authors believe that repetitive microtrauma is the reason why mature fat cells and fibroblasts of the epineurium hypertrophy.^2^, ^7^ Other authors believe that there is an unknown congenital abnormality that affects the growth of fibrofatty tissue.^10^

There are no pathognomonic findings for LFH.^2^ History of trauma, increasing pain, tenderness, diminished sensation, and paresthesia due to an enlarging mass causing compression neuropathy may be present. Carpal tunnel syndrome is a late complication that is observed in some cases.^1^, ^2^, ^4^, ^6^, ^7^, ^10^, ^11^, ^12^

The differential diagnoses of LFH include ganglionic cysts, hereditary hypertrophic neuritis of Dejerine‐Sottas syndrome, vascular malformations, traumatic neuromas, schwannomas, neurofibromas, intraneural lipomas, fibromatosis, and plexiform neurofibroma.^2^, ^7^ True lipomas can be differentiated from it because they are sharply demarcated, well‐encapsulated masses that normally occur on the surface; they arise in the perineurium or epineurium and contain no neural elements.^2^

Radiographs may only be helpful in viewing macrodactyly changes and soft tissue opacity.^12^ Diagnosis of LFH can be done using ultrasonography and MRI without the need for a biopsy. In fact, if there is familiarity with the features of the ultrasound, MRI and biopsy may be obviated.^13^ Its ultrasonographic findings show smooth, rounded, thickened hypoechoic or anechoic fascicles surrounded by echogenic fatty tissue; it commonly shows no flow on color Doppler.^14^ Additionally, fusiform nerve enlargement and thickened axonal bundles may be observed on MRI. Due to the epineural fibrous tissue that encapsulates the bundles, its axial images give a coaxial cable‐like and spaghetti‐like appearance.^15^ Electromyography and nerve conduction studies may also be useful to confirm compressive neuropathy.^14^

In LFH, diffuse involvement of the nerve showing relative bland changes is consistent with a hamartomatous rather than a neoplastic tumor. The lesion is described as a hamartoma because of the overgrowth of connective tissue constituents observed. The proportion of fibrous tissue to fat vary from case to case.^15^ Histologically, the lesions are characterized based on the connective and adipose mature tissue that surround and infiltrate the major nerve trunk and infiltrate the perineurium and epineurium. Considering these, its differential diagnoses include lipomatous neurofibroma, lipofibromatosis, diffuse lipomatosis, and intraneural perineurioma.^16^

Due to the context of a benign tumor, there are different recommendations for the treatment of LFH. These include observation, biopsy, intraneural neurolysis, and/or carpal tunnel release.^2^ In asymptomatic patients, different authors recommend observational treatment as the first option due to tumoral regression.^12^, ^17^ In symptomatic patients, there are different surgical procedures that may be considered. Carpal tunnel release alone can lower tumor size, decrease pain severity, and improve opposition strength.^16^, ^18^ However, it is important to note that patients treated with nerve excision and neurolysis may develop sensory deficits upon 2‐point discrimination testing.^6^, ^7^, ^9^, ^10^, ^11^

According to Mohamed et al^7^ the standard management currently being used by most surgeons is limited biopsy taking with carpal tunnel release. Furthermore, Amadio et al^8^ had 14 cases and concluded that microsurgical dissection was not clinically more successful than radical excision. Nanno et al^19^ reported a similar case in which the ulnar digital nerve of the thumb was affected; they performed limited resection of the tumor to prevent excessive damage to the nerve, and after a 3‐year follow‐up period, there was no recurrence of the mass nor any neurological deficits observed. Steentoft and Solerman^20^ had a similar case in which they used a microscope and partially resected the tumor without compromising the digital nerve; after a 2‐year follow‐up period, the patient had numbness of the index finger.

## 4 CONCLUSIONS

In conclusion, LFH is a rare condition with different clinical presentations. Evidence suggests that the diagnosis can be made with ultrasound and MRI without the need for a biopsy. In asymptomatic patients, treatment can be limited; however, if the tumor is symptomatic, surgical excision can be performed. In our case, the LFH was symptomatic, presented in an atypical location, and was not associated with macrodactyly; therefore, we decided to perform a complete excision. This pathology still needs further research to better understand its pathophysiology and to determine the best treatment option for it based on patient characteristics.

## CONFLICT OF INTEREST

None declared.

## AUTHOR CONTRIBUTIONS

JBS, RBF, and RASM were involved in patient care and wrote and reviewed the manuscript. LECC and LMBL reviewed the pathological images and provided histopathological research. All authors were involved in the editing and final approval of the manuscript.

## ETHICAL APPROVAL

Written informed consent was obtained from the patient for publication of this case report and any accompanying images. A copy of the written consent is available for review by the Editor in Chief of this journal.

## Data Availability

The data that support the findings of this study are available from the corresponding author upon reasonable request.
